# Plastome structure and phylogenetic relationships of Styracaceae (Ericales)

**DOI:** 10.1186/s12862-021-01827-4

**Published:** 2021-05-28

**Authors:** Xiu-Lian Cai, Jacob B. Landis, Hong-Xin Wang, Jian-Hua Wang, Zhi-Xin Zhu, Hua-Feng Wang

**Affiliations:** 1 Hainan Key Laboratory for Sustainable Utilization of Tropical Bioresources, College of Tropical Crops, Hainan University, Haikou, 570228 China; 2 School of Integrative Plant Science, Section of Plant Biology and the L.H. Bailey Hortorium, Cornell University, Ithaca, NY 14850 USA; 3 BTI Computational Biology Center, Boyce Thompson Institute, Ithaca, NY 14853 USA

**Keywords:** Styracaceae, Plastome, Genome structure, Phylogeny, Positive selection

## Abstract

**Background:**

The Styracaceae are a woody, dicotyledonous family containing 12 genera and an estimated 160 species. Recent studies have shown that *Styrax* and *Sinojackia* are monophyletic, *Alniphyllum* and *Bruinsmia* cluster into a clade with an approximately 20-kb inversion in the Large Single-Copy (LSC) region. *Halesia* and *Pterostyrax* are not supported as monophyletic, while *Melliodendron* and *Changiostyrax* always form sister clades. *Perkinsiodendron* and *Changiostyrax* are newly established genera of Styracaceae. However, the phylogenetic relationship of Styracaceae at the generic level needs further research.

**Results:**

We collected 28 complete plastomes of Styracaceae, including 12 sequences newly reported here and 16 publicly available sequences, comprising 11 of the 12 genera of Styracaceae. All species possessed the typical quadripartite structure of angiosperm plastomes, with sequence differences being minor, except for a large 20-kb (14 genes) inversion found in *Alniphyllum* and *Bruinsmia*. Seven coding sequences (*rps4*, *rpl23*, *accD*, *rpoC1*, *psaA*, *rpoA* and *ndhH*) were identified to possess positively selected sites. Phylogenetic reconstructions based on seven data sets (i.e., LSC, SSC, IR, Coding, Non-coding, combination of LSC + SSC and concatenation of LSC + SSC + one IR) produced similar topologies. In our analyses, all genera were strongly supported as monophyletic. *Styrax* was sister to the remaining genera. *Alniphyllum* and *Bruinsmia* form a clade. *Halesia diptera* does not cluster with *Perkinsiodendron*, while *Perkinsiodendron* and *Rehderodendron* form a clade. *Changiostyrax* is sister to a clade of *Pterostyrax* and *Sinojackia*.

**Conclusion:**

Overall, our results demonstrate the power of plastid phylogenomics in improving estimates of phylogenetic relationships among genera. This study also provides insight into plastome evolution across Styracaceae.

**Supplementary Information:**

The online version contains supplementary material available at 10.1186/s12862-021-01827-4.

## Background

The Styracaceae DC. & Spreng (Ericales) comprise an angiosperm clade of 12 genera and over 160 species, mainly distributed in regions of Asia, as well as tropical and temperate America, and the Mediterranean [1]. The family consists of shrubs or trees, usually having stellate pubescent or epidermal scales, simple leaves, with raceme, cyme or panicle inflorescence, and actinomorphic flowers with varying degrees of synsepaly and sympetaly [2]. The fruit of Styracaceae is a drupe or capsule, with persistent calyx, surrounding or united with the fruit. The Styracaceae have been included in a number of morphological studies, analyzing leaf anatomy [3], wood anatomy [4], pollen morphology [5] and floral morphology and anatomy [2], but distinguishing between genera in the family primarily involves variation in fruit morphological characters (e.g. hypanthium at maturity). On one hand the ovary is inferior with a persistent hypanthium combined with the fruit at maturity [(i.e., *Changiostyrax* C.T. Chen (one species), *Halesia* J. Ellis ex L (two species), *Melliodendron* Hand.-Mazz (one species), *Parastyrax* Siebold & Zucc. (two species), *Perkinsiodendron* P. W. Fritsch (one species), *Pterostyrax* W.W. Sm*.*(four species), *Rehderodendron* Hu (one species), and *Sinojackia* Hu (seven species)]. On the other hand, the ovary is superior and a persistent hypanthium forms only at the base of the fruit at maturity [*Alniphyllum* Matsum (three species), *Bruinsmia* Boerl. & Koord (two species), and *Styrax* L (130 species)]. Moreover, the ovary of *Huodendron* Rehder (four species) is semisuperior with a persistent hypanthium extending from the base to about two-thirds of the fruit length [1, 2], a feature considered to be transitional.

The systematic position of Styracaceae and the genera within have been unstable since the establishment of the family by Dumoritor in 1829 [6]. Early researchers thought Styracaceae was positioned in the order Ebenales, along with the well-known Sapotaceae, Ebenaceae, and Symplocaceae, and the small family Lissocarpaceae [7–10]. However, Cronquist [10] showed that these families have some original characteristics and some new evolutionary characters, which may have arisen via parallel evolution. Based on embryological and anatomical studies, Herbert [11] suggested that Styracaceae and Theaceae may have originated from a common ancestor, since the two share many common characteristics. According to molecular systematic studies, Styracaceae has been recognized as part of the order Ericales sensu lato [12].

Within the family, phylogenetic resolution generally remains poor. At most 17 genera have been included in Styracaceae, with *Symplocos* L, *Diclidanthera* Mart*, Afrostyrax* Perk et Gil, *Foveolaria* Ruiz et pav*.*, *Pamphilia* Mart. ex A. DC, *Huapierre* et De Wil, and *Lissocarpa* Benth placed in the Styracaceae by various authors [13]. *Symplocos*, *Diclidanthera*, and *Lissocarpa* were excluded from Styracaceae by Perkins [14]*. Symplocos* was treated as an independent family (Symplocaceae Desf) [15]. *Diclidanthera* was placed in Polygalaceae [7, 15], and *Lissocarpa* was placed in Ebenaceae [16]. *Afrostyrax* was once included in the genus *Styrax* [17], but was later reclassified into Huaceae [7, 15, 18]. According to taxonomic revisions, *Pamphilia* was classified into *Styrax* [19], while Fritsch [20] combined *Foveolaria* into *Styrax* by implementing morphological phylogenetic analyses. In addition, two new genera have been established: (1) Chen [21] segregated *Sinojackia dolichocarpa* as a new monotypic genus *Changiostyrax*, and (2) according to morphological and DNA sequences, *Halesia macgregorii* was removed from *Halesia* to become a new genus, *Perkinsiodendron* P.W. Fritsch [22].

Although the phylogenetic placement of the family has been resolved, relationships between genera remain ambiguous. The phylogeny of Ericales based on the chloroplast gene *rbcL* [23] suggested that Styracaceae was polyphyletic with *Styrax* and *Clethra* Gronov. ex L*.* (Clethraceae) clustered in a clade, while *Halesia*, *Rehderodendron*, and *Sinojackia* formed a clade that was sister to *Diapensia* L*.* and *Galax* Rafin*.* (Diapensiaceae). However, the interpretation of polyphyly does not always hold true. Olmstead et al. [24] inferred the phylogeny of Asteridae based on the chloroplast gene *ndhF*, showing a strongly supported sister relationship between *Styrax* and *Halesia*. Albach et al. [25] came to the same conclusion based on the DNA gene sequences *atpB*, *ndhF*, *rbcL* and 18S [24] within the Asterids. In addition, the phylogeny of Styracaceae based on morphology plus three DNA sequences (chloroplast *trnL intron*/*trnL-trnF* spacer and *rbcL* with the nuclear ribosomal DNA region ITS) recovered a monophyletic relationship of Styracaceae [1]. *Pterostyrax* and *Halesia* were not supported as monophyletic, since *Styrax* and *Huodendron* formed a clade that was sister to a clade of *Alniphyllum* and *Bruinsmia*, and a sister relationship was found between *Halesia macgregorii* and *Rehderodendron macrocarpum* [1]. Based on ITS, the plastid *psbA-trnH* intergenic spacer, and microsatellite data, Yao et al. [26] recovered *Sinojackia* as monophyletic and reported a similar topology as Fritsch et al. [1] with weak support for six genera within Styracaceae. Yan et al. [27] conducted phylogenetic analyses of Styracaceae based on 19 chloroplast genomes. The results showed that *Styrax* was monophyletic, while *Alniphyllum* and *Bruinsmia* clustered in a clade with an approximate 20-kb inversion in the Large Single-Copy (LSC) region. Species of *Pterostyrax* were not supported as monophyletic, with *Halesia carolina* L and *Pterostyrax hispidus* Siebold & Zucc forming a clade.

The chloroplast genomes of most angiosperms are maternally inherited. The rate of evolution of genes in the chloroplast is relatively slow overall, but differences have been observed across different regions of the plastome, which can be applied to phylogenetic studies of various taxonomic scales. Signatures of selection (purifying or positive/adaptive) have been observed in different regions of the plastome, including protein coding regions involved in photosynthesis [28–30]. Several aspects have led to the extensive use of plastomes for phylogenetic inference such as a conserved structure, small effective population size, and lack of recombination due to being predominately uniparentally inherited [31–33]. With the increasing efficiency of next-generation sequencing (NGS) technologies, obtaining whole-plastome sequence data has become cheaper and easier. Whole-plastomes have been used in taxonomically complex groups to generate resolved and well-supported phylogenies, as well as serving as sequence barcodes to identify plant species at the molecular level [34–36].

Despite progress in understanding phylogenetic relationships within Styracaceae, most advances have been based on relatively limited molecular and/or morphological data. Only one study has examined the phylogeny of Styracaceae using plastome-scale data [27], but this study employed only 19 taxa and included only one or two accessions per genus. Here, we increased sampling for some genera, especially *Sinojackia* (five accessions) and *Styrax* (seven accessions). We analyzed 28 complete plastomes for resolving the broader phylogeny of Styracaceae. Compared with phylogenetic studies limited to a few complete plastomes or a few plastid loci, plastome phylogenomic studies provide potentially greater resolution and support. The objectives of this study are: (1) infer the plastome structural evolution of Styracaceae, (2) resolve the phylogenetic relationships of Styracaceae, (3) use selective pressure analysis to test for the presence of adaptive evolution in all genes.

## Methods

### Plant samples, DNA extraction, sequencing and assembly

We collected 28 plastomes of Styracaceae, including 12 newly sequenced and 16 previously sequenced plastomes (Table 1), with representatives from 11 of the 12 genera described by APG IV [37]. We used *Symplocos ovatilobata* Noot (Symplocaceae), *Stewartia monadelpha* Siebold et Zucc, and *Stewartia sinii* (Y. C. Wu) Sealy (Theaceae) as outgroups. A total of 31 sequences were analyzed. Our field collections were permitted by the government following local ethics and laws. Collected plant leaves were put directly into silica gel to dry. The formal identification of the plant material was undertaken by Guowen Xie, and voucher herbarium specimens were deposited at the Institute of Tropical Agriculture and Forestry (HUTB), Hainan University, Haikou, China.

**Table 1:** Plant collection information and GenBank accession numbers for plastomes of Styracaceae and outgroups included in this study

Family	Species name	Specimen collection and voucher specimen	Locality	Accession number
Styracaceae	*Alniphyllum eberhardtii*	Yan M.H. 201,401 (HIB)	Kunming Institute of Botany,China	NC_031892_1
Styracaceae	*Alniphyllum fortunei*	HUTB LC	Lushan Mountain, Jiujiang, Jiangxi	MT700470
Styracaceae	*Styrax grandiflorus*	NA	Yunnan, China	NC_030539_1
Styracaceae	*Alniphyllum pterospermum*	NA	Wuhan,Hubei,China	NC_041126_1
Styracaceae	*Bruinsmia polysperma*	Wang Hong 9805 (HIB)	Pu'er, Jinggu County, Yunnan, China	NC_030180_1
Styracaceae	*Bruinsmia styracoides*	P.W. Fritsch 1886 (CAS)	Sabah, Malaysia	NC_041137_1
Styracaceae	*Changiostyrax dolichocarpa*	HUTB SZ1	Hupingshan, Hunan, China	MT700471
Styracaceae	*Changiostyrax dolichocarpa*	HUTB SZ2	Hupingshan, Hunan, China	MT700472
Styracaceae	*Halesia diptera*	P.W. Fritsch 1975 (CAS)	University of California Botanical Garden, California,	NC_041128_1
Styracaceae	*Halesia_carolina*	P.W. Fritsch 1974 (CAS)	University of California Botanical Garden, California,	NC_041127_1
Styracaceae	*Huodendron biaristatum*	Yan M.H. 201,403 (HIB)	Wuhan Botanical Garden, Hubei, China	NC_041132_1
Styracaceae	*Melliodendron xylocarpum*	YXQ138	NA	MF179500_1
Styracaceae	*Perkinsiodendron macgregorii*	Zhao C.X. 201,401 (HIB)	Nanyue Arboretum, Hunan, China	MG719841_1
Styracaceae	*Pterostyrax corymbosus*	Yan M.H. 201,405 (HIB)	Wuhan Botanical Garden, Hubei, China	NC_041134_1
Styracaceae	*Pterostyrax hispidus*	P.W. Fritsch 1970 (CAS)	Quarryhill Botanical Garden, California, U.S.A	NC_041135_1
Sstyracaceae	*Pterostyrax psilophyllus*	Yan M.H. 201,406 (HIB)	Wuhan Botanical Garden, Hubei, China	NC_041133_1
Styracaceae	*Rehderodendron macrocarpum*	Zhao C.X. 201,402 (HIB)	Nanyue Arboretum, Hunan, China	NC_041139_1
Styracaceae	*Sinojackia microcarpa*	HUTB B274	Jiande, Zhejiang, China	MT700474
Styracaceae	*Sinojackia rehderiana*	HUTB PZ13	Pengze, Jiangxi,China	MT700475
Styracaceae	*Sinojackia sarcocarpa*	HUTB B242	Leshan, Sichuan,China	MT700476
Styracaceae	*Sinojackia sarcocarpa*	HUTB B243	Sichuan Normal University,China	MT700477
Styracaceae	*Sinojackia xylocarpa*	HUTB NJ	Nanjing, Botanical, Garden, Jiangsu,China	MT700481
Theaceae	*Stewartia monadelpha*	S. Sakaguchi s. n	Nara, Kinki, Japan	NC_041468_1
Theaceae	*Stewartia sinii*	H. Y. Lin 16,105	Jinxiu Co., Guangxi, China	NC_041470_1
Styracaceae	*Styrax confusus*	HUTB SS	Lushan Mountain, Jiujiang, Jiangxi	MT700478
Styracaceae	*Styrax faberi*	HUTB B197	Lushan Mountain, Jiujiang, Jiangxi	MT700480
Styracaceae	*Styrax ramirezii*	P. W. Fritsch 1472 (CAS)	University of California Botanical Garden, California,U.S.A	NC_041138_1
Styracaceae	*Styrax suberifolius*	Zhao C.X. 201,403 (HIB)	Nanyue Arboretum, Hunan, China	NC_041125_1
Styracaceae	*Styrax zhejiangensis*	NA	NA	NC_038209_1
Styracaceae	*Styrax dasyanthus*	HUTB CH	Lushan Mountain, Jiujiang, Jiangxi	MT700479
Symplocaceae	*Symplocos ovatilobata*	HUTB	Diaoluo Mountain,Hainan, China	NC_036489_1

Total genomic DNA was extracted from dried leaf tissue using cetyltrimethyl ammonium bromide (CTAB) protocol of Doyle and Doyle [38]. Genomic DNA of each sample was quantified and analyzed with an Agilent BioAnalyzer 2100 (Agilent Technologies). Samples yielding at least 0.8 µg DNA were selected for subsequent library construction and sequencing. Genomic DNA of selected samples was used to build paired‐end libraries with insert sizes of 200–400 bp according to the manufacturer’s instructions [39]. Sequencing of the new 12 accessions was completed using BGISEQ-500 2 × 100 at BGI (Shenzhen, China). This yielded approximately eight Gb of high‐quality data per sample of 100 bp paired‐end reads. Raw reads were trimmed using SOAPfilter v2.2 (BGI-Shenzhen, China) with the following criteria: removal of reads with more than 10 percent base of N, reads with more than 40 percent low quality (phred score less than 10), and reads contaminated by adaptors and PCR duplicates. Approximately six Gb of clean data (high-quality reads > phred score35) were obtained for each sample. For all samples, plastomes were assembled using MITObim v1.8 [40] with default parameters and using plastomes of related species as templates for assembly (Table 2). The assembly was ordered using BLAST and aligned (> 90% similarity and query coverage) according to the reference chloroplast genome (Table 2). To verify sequencing depth and contig overlap, cleaned reads were mapped to reference plastomes in Geneious R11.0.4 [41].

**Table 2:** GenBank accession numbers, and template plastome for assembly for 12 newly sequenced genomes

Family	Species name	Accession number	Locality	Template for plastome assembly
Styracaceae	*Alniphyllum fortunei (Hemsl.) Makino*	MT700470	Lushan Mountain, Jiujiang, Jiangxi	KX765434.1
Styracaceae	*Pterostyrax corymbosus Sieb. et Zucc*	MT700473	Lushan Mountain, Jiujiang, Jiangxi	KY709672.1
Styracaceae	*Changiostyrax dolichocarpa*	MT700471	Hupingshan,Hunan,China	MF179499.1
Styracaceae	*Changiostyrax dolichocarpa*	MT700472	Hupingshan,Hunan,China	MF179499.1
Styracaceae	*Sinojackia rehderiana Hu*	MT700475	Pengze, Jiangxi,China	MF179499.1
Styracaceae	*Sinojackia xylocarpa Hu*	MT700481	Nanjing Botanical Garden, Jiangsu,China	KY709672.1
Styracaceae	*Sinojackia microcarpa C.T. Chen & G. Y. Li*	MT700474	Jiande,Zhejiang, China	KY626040.1
Styracaceae	*Sinojackia sarcocarpa L. Q. Luo*	MT700476	Sichuan Normal University,China	KY709672.1
Styracaceae	*Sinojackia sarcocarpa L. Q. Luo*	MT700477	Leshan, Sichuan,China	KY709672.1
Styracaceae	*Styrax confusus Hemsl*	MT700478	Lushan Mountain, Jiujiang, Jiangxi	MF179493.1
Styracaceae	*Styrax dasyanthus Perk*	MT700479	Lushan Mountain, Jiujiang, Jiangxi	MF179493.1
Styracaceae	*Styrax faberi Perkins Wenzhou*	MT700480	Lushan Mountain, Jiujiang, Jiangxi	KX111381.1

### Genome annotation

Plastomes were annotated using Geneious R11.0.4 [41] using the same reference plastomes used for assembly. Start/stop codons and intron/exon boundaries were further corrected using Dual Organellar GenoMe Annotator (DOGMA) [42]. In addition, tRNAscan-SE1.21 was used to further verify all tRNA genes. We also re-annotated the downloaded assembled plastomes from previous studies before using them in our analyses. The 12 newly generated complete plastome sequences were deposited in GenBank (Accession Numbers in Table 2).

### Genome comparative and structural analyses

Graphical maps of Styracaceae plastomes were drawn using Organellar Genome DRAW (OGDRAW) [43], with subsequent manual editing. Genome comparisons across the 26 Styracaceae species (selecting one sequence per species) were performed with Shuffle-LAGAN mode in mVISTA [44] using the annotation of *Pterostyrax hispidus* Siebold & Zucc as a reference. To evaluate whether different chloroplast genome regions have undergone different evolutionary histories and to explore highly variable regions for future population genetic and species identification studies, we sequentially extracted both coding regions and noncoding regions (including intergenic spacers and introns) after aligning with MAFFT v7 [45] under the criteria that the aligned length was > 200 bp and at least one mutation site was present. Finally, nucleotide variability of these regions was evaluated with DNASP v5.10 [46].

### Selective pressure analysis

The analyses of selective pressures were conducted along the phylogenetic tree of Styracaceae (see below) for each plastid gene located in the Large Single-Copy (LSC) region, Inverted Repeat (IR) region and Small Single-Copy (SSC) region. Nonsynonymous (dN) and synonymous (dS) substitution rates of each plastid gene were calculated using the yn00 program in PAML v4.9 [47]. In addition, we used the CODEML program in PAML to detect signatures of natural selection among specific lineages. Genes were considered to be under positive/negative selection at a certain clade when its ω value from the two-ratio model was higher/lower than 1 (neutral selection). To avoid potential convergence biases, genes with too few mutations [*Pi*(nucleotide diversity) < 0.001] were filtered out from selective pressure analysis.

### Phylogenetic analyses

Phylogenetic analyses were conducted on the 31 plastomes, using *Symplocos ovatilobata, Stewartia sinii,* and *S. monadelpha* as outgroups. Chloroplast sequences were aligned using MAFFT v7.037 [45]. To evaluate possible alternative phylogenetic hypotheses, topologies were constructed by both maximum likelihood (ML) and Bayesian inference (BI) methods using not only the complete genome sequences, but also using seven additional data sets (i.e. LSC, SSC, IR, Coding, Noncoding, combination of LSC + SSC, and concatenation of LSC + SSC + one IR). Data characteristics and the best-fitting models of nucleotide substitutions were determined with Akaike Information Criterion (AIC) in Modeltest v3.7 [48] (Table 3). For the coding data set, PartitionFinder v2.1.1 [49] was used to select the best-fit partitioning scheme of all 79 possible gene-by-codon position partitions (79 genes × 3 codon positions).

**Table 3:** Data characteristics and models selected in Maximal Likelihood and Bayes Inference analyses for phylogenetic data sets

Datasets	No. of taxa	No. of site	No. of variable (%)	Parsimony informative sites (%)	Best Fit Model	Model in ML	Model in BI
Whole plastomes	31	180369	31865 (17.66)	21804 (12.08)	GTR + I + G	GTR + I + G	TVM + I + G
Coding	31	79755	13242 (16.60)	9395 (11.78)	GTR + I + G	GTR + I + G	GTR + I + G
Noncoding	31	131319	21014 (16.00)	11940 (9.09)	TVM + I + G	GTR + I + G	TVM + I + G
IRb	31	28419	1900 (6.68)	938 (3.30)	TVM + I + G	GTR + I + G	TVM + I + G
LSC	31	104030	23519 (22.60)	17151 (16.49)	GTR + I + G	GTR + I + G	GTR + G
SSC	31	22329	5021 (22.49)	3024 (13.54)	TVM + I + G	GTR + I + G	GTR + I + G
LSC + SSC	31	126237	28623 (22.67)	20158 (15.96)	GTR + I + G	GTR + I + G	GTR + I + G

IR, Inverted repeat; LSC, Large single copy; SSC, Small single copy

Maximum likelihood analyses were conducted using RAXML-HPC v8.2.8 [50] with 1000 bootstrap replicates on the CIPRES Science Gateway website [51] with the GTR + I + G substitution model. Bayesian inference (BI) analyses were performed in MrBayes v3.2 [52] on the CIPRES Science Gateway portal [51] with the following conditions used for the protein-coding dataset: starting from random trees, Markov chain Monte Carlo (MCMC) simulations were ran for 900,000,000 generations with four incrementally heated chains sampling every 1000 generations. BI analyses were set up identically for the remaining data sets, except that 50,000,000 generations were simulated. Convergence of the MCMC chains was determined by examining the average standard deviation of the split frequencies (< 0.01). The first 25% of the trees were discarded as burn-in. The effective sample size (ESS > 200) was determined by using Tracer v 1.7 [53].

## Result

### Plastome structure of styracaceae

**Fig. 1:**
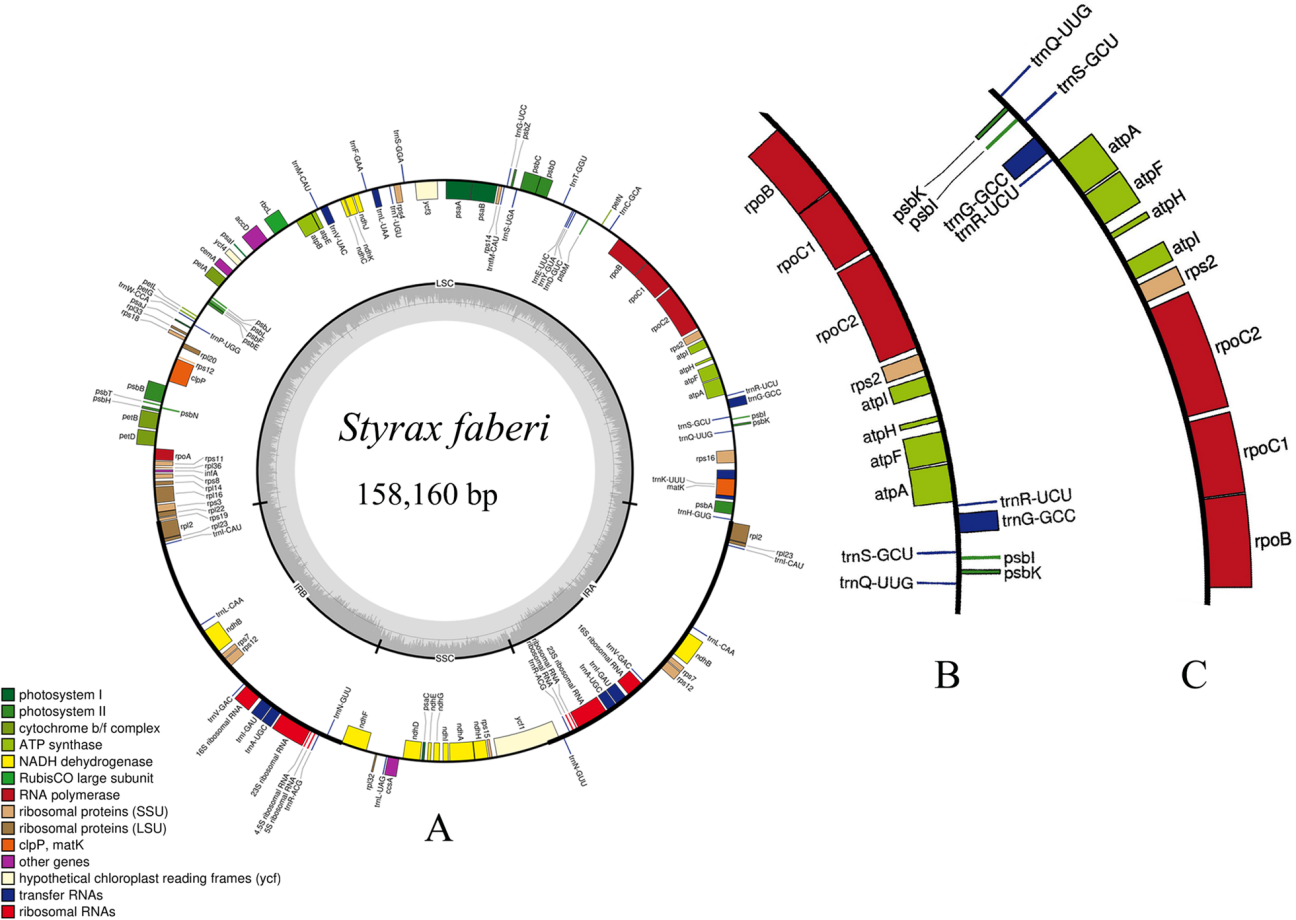
Gene map of the *Styrax faberi.* **A** The inverted order of genes in *Alniphyllum fortunei*; **B** The corresponding region of *Styrax faberi*

In this study, the plastomes of Styracaceae and outgroups displayed a typical quadripartite structure and similar lengths. Plastome sizes ranged from 155,185 bp (*Alniphyllum pterospermu* Matsum) to 158,879 bp (*Pterostyrax hispidus*) with a minimum/maximum read depth of 10 × /40 × for each plastome. The plastomes were composed of a large single-copy (LSC) region (ranging from 83,200 bp to 88,258 bp), a small single-copy (SSC) region (ranging from 17,556 bp to 19,235 bp), and two inverted repeat IR regions (IRa and IRb) (ranging from 24,243 bp to 26,761 bp) (Table 4). Their overall GC content was nearly identical (36.70–37.40%). In all species, the GC content of the LSC and SSC regions (about 35% and 30%) were lower than those of the IR regions (about 43%). The 31 plastomes encoded 113 genes, including 79 protein-coding genes, 30 transfer RNA (tRNA) genes, and four ribosomal RNA (rRNA) genes. Comparison of the genome structures among Styracaceae, revealed an inversion of a large segment spanning *trnQ-UUG* to *rpoB* (20-kb) in the LSC region of *Alniphyllum fortunei* (Hemsl.) Makino (Fig. 1).

**Table 4:** Summary of major plastome characteristics in Styracaceae and outgroups

Latin name	cpDNA size (bp)	LSC size (bp)	SSC size (bp)	IRs size (bp)	Total GC content (%)	LSC (%)	SSC (%)	IR (%)	tRNA	rRNA	Coding gene	Number
*Alniphyllum eberhardtii*	155384	83710	18153	26761	37.10	35.20	30.20	42.40	30	4	79	NC_031892_1
*Alniphyllum fortunei*	155490	83773	18153	26782	37.10	35.20	30.20	42.40	30	4	79	MT700470
*Alniphyllum pterospermum*	155185	83200	18583	26701	37.10	35.20	30.10	42.50	30	4	79	NC_041126_1
*Bruinsmia polysperma*	157879	86495	18725	26329	36.80	34.90	30.30	42.20	30	4	79	NC_030180_1
*Bruinsmia styracoides*	156434	86251	19235	25574	36.70	34.80	29.80	42.60	30	4	79	NC_041137_1
*Changiostyrax dolichocarpa*	158881	88086	18609	26091	37.30	35.30	30.50	43.00	30	4	79	MT700471
*Changiostyrax dolichocarpa*	158781	88030	18606	26072	37.30	35.30	30.50	43.00	30	4	79	MT700472
*Halesia diptera*	158849	88165	18528	26078	37.20	35.20	30.50	43.00	30	4	79	NC_041128_1
*Huodendron biaristatum*	158499	87731	18988	25990	36.80	34.70	30.30	42.70	30	4	79	NC_041132_1
*Melliodendron xylocarpum*	157131	90159	18486	24243	37.20	35.30	30.60	43.20	30	4	79	MF179500_1
*Perkinsiodendron macgregorii*	158602	88189	18293	26060	37.20	35.20	30.60	43.00	30	4	79	MG719841_1
*Pterostyrax corymbosus*	158836	88102	18557	26088	37.20	35.20	30.50	43.00	30	4	79	NC_041134_1
*Pterostyrax corymbosus*	158890	85662	18561	26106	37.20	35.30	30.50	43.10	30	4	79	MT700473
*Pterostyrax hispidus*	158879	88195	18516	26087	37.20	35.20	30.50	43.00	30	4	79	NC_041135_1
*Pterostyrax psilophyllus*	158835	88101	17556	26089	37.20	35.20	30.50	43.00	30	4	79	NC_041133_1
*Rehderodendron macrocarpum*	157934	87508	18316	25368	37.20	35.20	30.60	43.00	30	4	79	NC_041139_1
*Sinojackia microcarpa*	157554	87142	18238	26089	37.30	35.30	30.70	43.00	30	4	79	MT700474
*Sinojackia rehderiana*	158872	88077	18516	26091	37.20	35.20	30.50	43.00	30	4	79	MT700475
*Sinojackia sarcocarpa*	158901	88168	18556	26090	37.20	35.20	30.50	43.00	30	4	79	MT700476
*Sinojackia sarcocarpa*	158834	88092	18881	25931	37.20	35.20	30.60	43.10	30	4	79	MT700477
*Sinojackia xylocarpa*	158637	87947	18552	26068	37.20	35.20	30.50	43.00	30	4	79	MT700481
*Stewartia monadelpha*	158447	87545	18134	26378	37.30	35.30	30.50	42.80	30	4	79	NC_041468_1
*Stewartia sinii*	158478	87531	18962	26363	37.30	35.30	30.60	42.80	30	4	79	NC_041470_1
*Styrax confusus*	158261	87837	18299	26064	37.00	34.80	30.30	42.90	30	4	79	MT700478
*Styrax faberi*	158160	87785	18225	26073	36.90	34.80	30.20	42.90	30	4	79	MT700480
*Styrax grandiflorus*	158052	87648	18310	26047	36.90	34.80	30.20	42.90	30	4	79	NC_030539_1
*Styrax ramirezii*	158315	87990	18051	26363	37.00	34.80	30.40	43.00	30	4	79	NC_041138_1
*Styrax suberifolius*	158480	87763	18051	26363	37.00	34.80	30.30	42.80	30	4	79	NC_041125_1
*Styrax zhejiangensis*	157387	87195	17988	25953	37.00	34.80	30.30	42.80	30	4	79	NC_038209_1
*Styrax dasyanthus*	158165	87736	18960	25736	36.90	34.80	30.30	43.00	30	4	79	MT700479
*Symplocos ovatilobata*	157417	87447	17792	26089	37.40	35.40	30.80	43.00	30	4	79	NC_036489_1

### Comparative genomic analysis and divergence hotspot regions

**Fig. 2:**
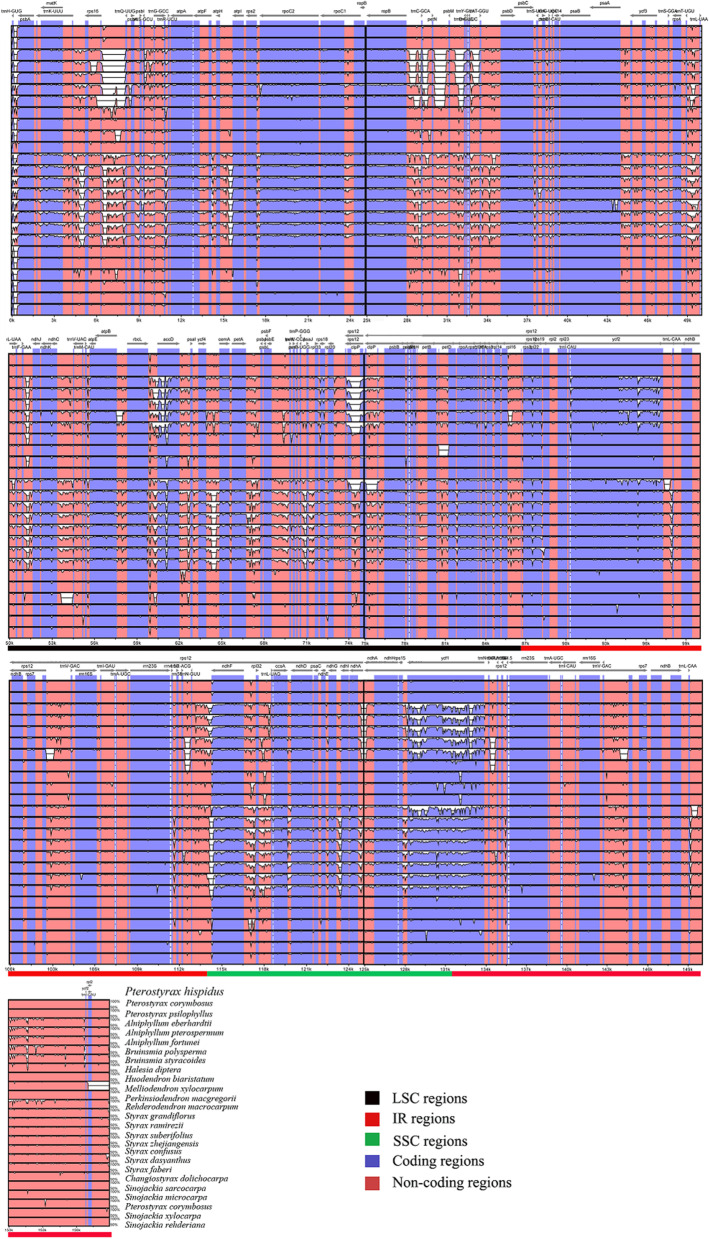
Visualization of the alignment of 26 Styracaceae plastome sequences. The plastome of *Pterostyrax hispidus* was used as the reference. The Y-axis depicts percent identity to the reference genome (50–100%) and the X-axis depicts sequence coordinates within the plastome. Genome regions were color-coded according to coding and noncoding regions

To investigate the levels of sequence divergence, 26 Styracaceae plastomes were plotted using mVISTA with *Pterostyrax hispidus* as the reference (Fig. 2). The sequence divergence was low among all plastomes. Notably, the proportion of variability in coding regions and inverted repeats (IRs) showed higher conservation than noncoding and small single-copy (SSC) regions. The mutation rate of *ycf1* was the highest observed. The variation rates of *Styrax* and *Huodendron* in the large and small single copy regions were higher than other species, and the sequence divergence of *Huodendron* in *clpP* intron lower than 50%.

**Fig. 3:**
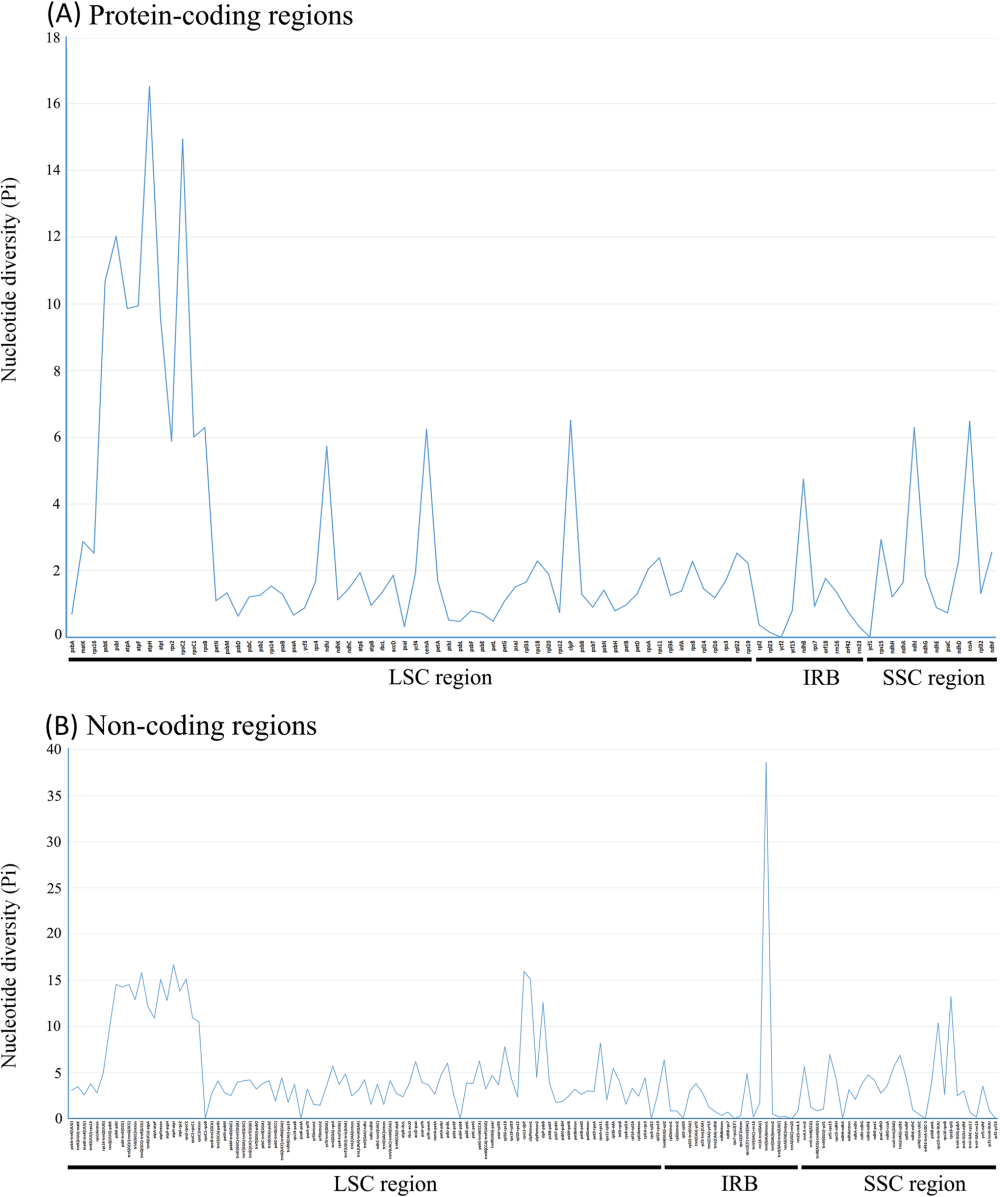
Comparison of the nucleotide diversity (Pi) values across 28 Styracaceae plastomes. **A** Protein-coding regions. **B** Noncoding regions

Nucleotide diversity (*pi*) analyses showed that the proportion of variable sites in noncoding regions were higher than that in coding region, and the greatest diversity change was in the intergenic spacer region (Fig. 3). Among all 209 loci (79 coding genes and 130 noncoding regions), nucleotide diversity values of coding genes ranged from 0.001 (*rpl23*) to 0.156 (*atpH*), with four loci greater than 0.1 (*psbK*, *psbI*, *rpoC2*, *atpH*). Nucleotide diversity of noncoding genes ranged from 0 (*rpoC1-rpoB*, *psaB-psaA*, *psbF-psbE*, *rps3-rpl22*, *rpl2-rpl23*, *rps7-rps12*, *trnA* (UGC)*-rrn23*, *ndhH-ndhA*, *orf42-trnA-UGC*, *ycf2-ycf15)* to 0.385 (*trnI* intron1). Seven loci possessed values > 0.15: e.g. *atpF* intron (0.151), *clpP* intron1 (0.151), *rps*2-*rpoC*2 (0.151), *trnG*(*GCC*)-*trnR*(*UCU*) (0.158), *rps*12-*clpP* (0.159), *atpH*-*atpI* (0.166), *trnI*(*GAU*) intron1 (0.385) (Fig. 3).

### Selective pressures in plastome evolution of Styracaceae

**Fig. 4:**
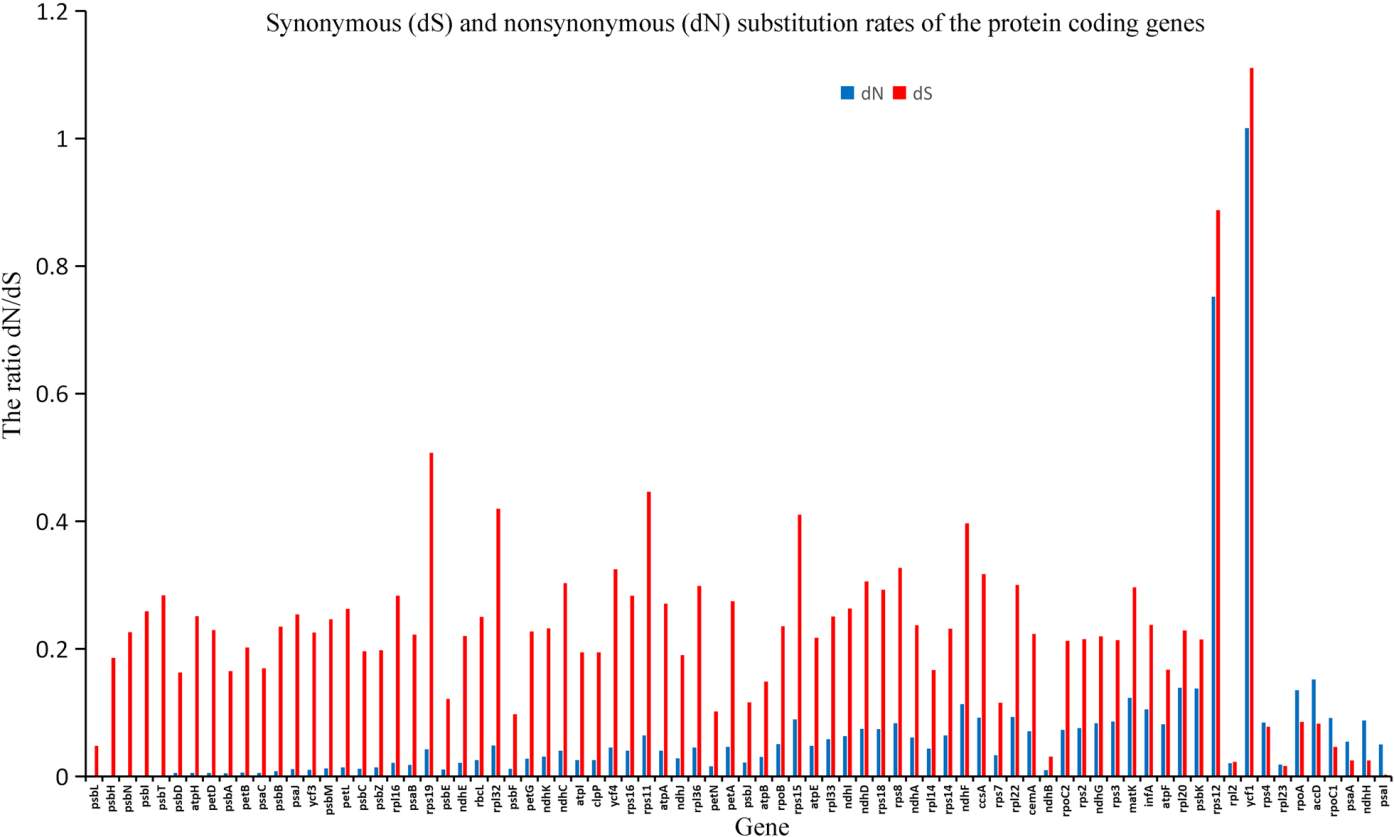
Synonymous (dS) and nonsynonymous (dN) substitution rates of the protein coding genes

**Fig. 5:**
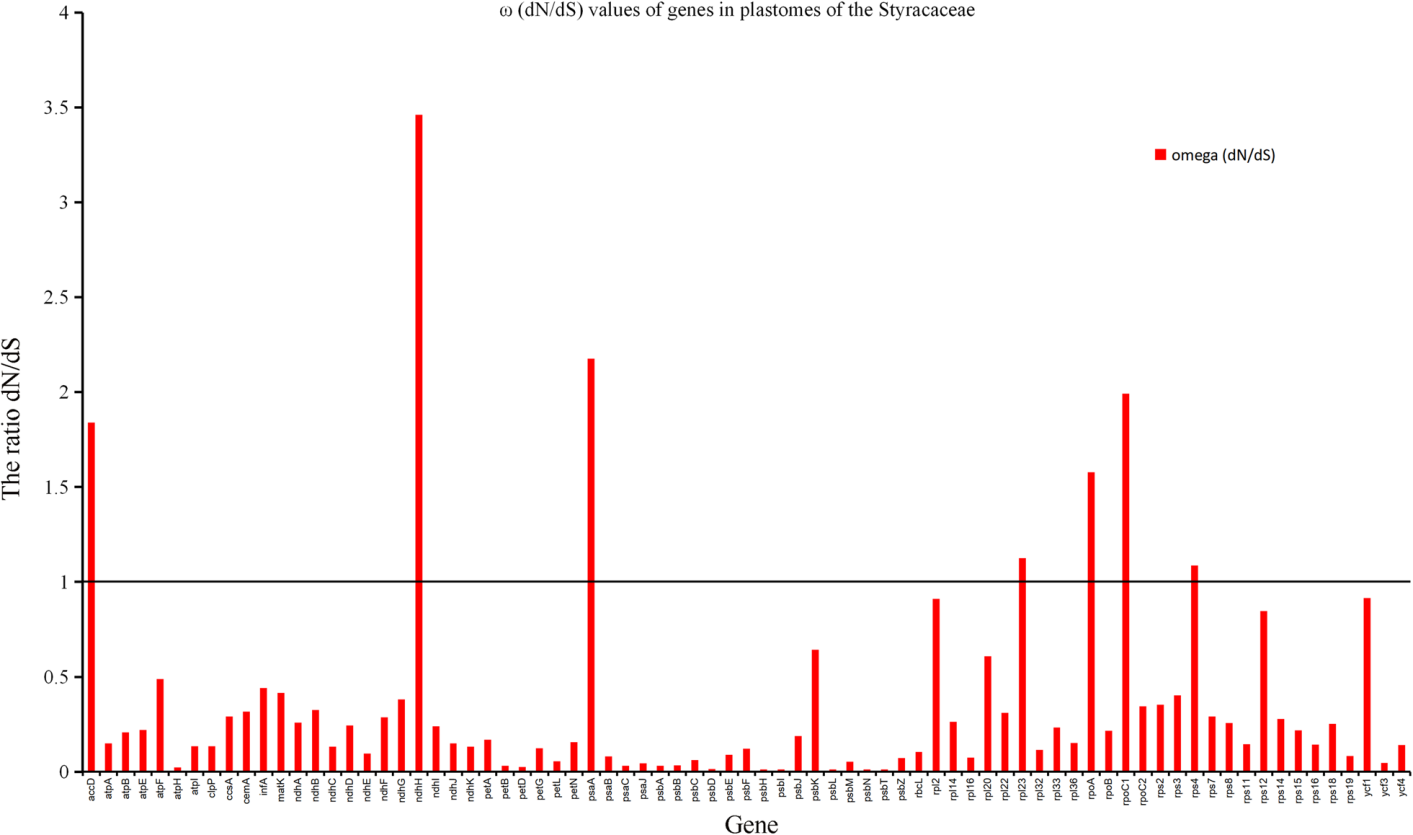
ω (dN/dS) values of genes in plastomes of the Styracaceae. The red line represents neutral selection, while values above one represents positive/adaptative selection, and values below one represents negative/purifying selection

The results showed that the 79 protein coding genes mainly possessed synonymous substitutions (Fig. 4). In addition, *rps12* (0.8874), *rps19* (0.5076) and *rps11* (0.4466) had the highest synonymous substitution rate. The locus with the highest rate of nonsynonymous substitution was *ycf1* (1.016). The rate of nonsynonymous substitutions in other genes was low, in which the rate of nonsynonymous substitution of *psb* was the lowest, and the nonsynonymous substitution of *psbL*, *psbH*, *psbN*, *psbI* and *psbT* was zero. Among the 79 protein coding genes of Styracaceae, there were seven genes with ω value greater than 1: *rps4* (1.087), *rpl23* (1.126), *accD* (1.839), *rpoC1* (1.990), *psaA* (2.175), *rpoA* (1.578) and *ndhH* (3.459) (Fig. 5).

### Phylogenetic analyses

**Fig. 6:**
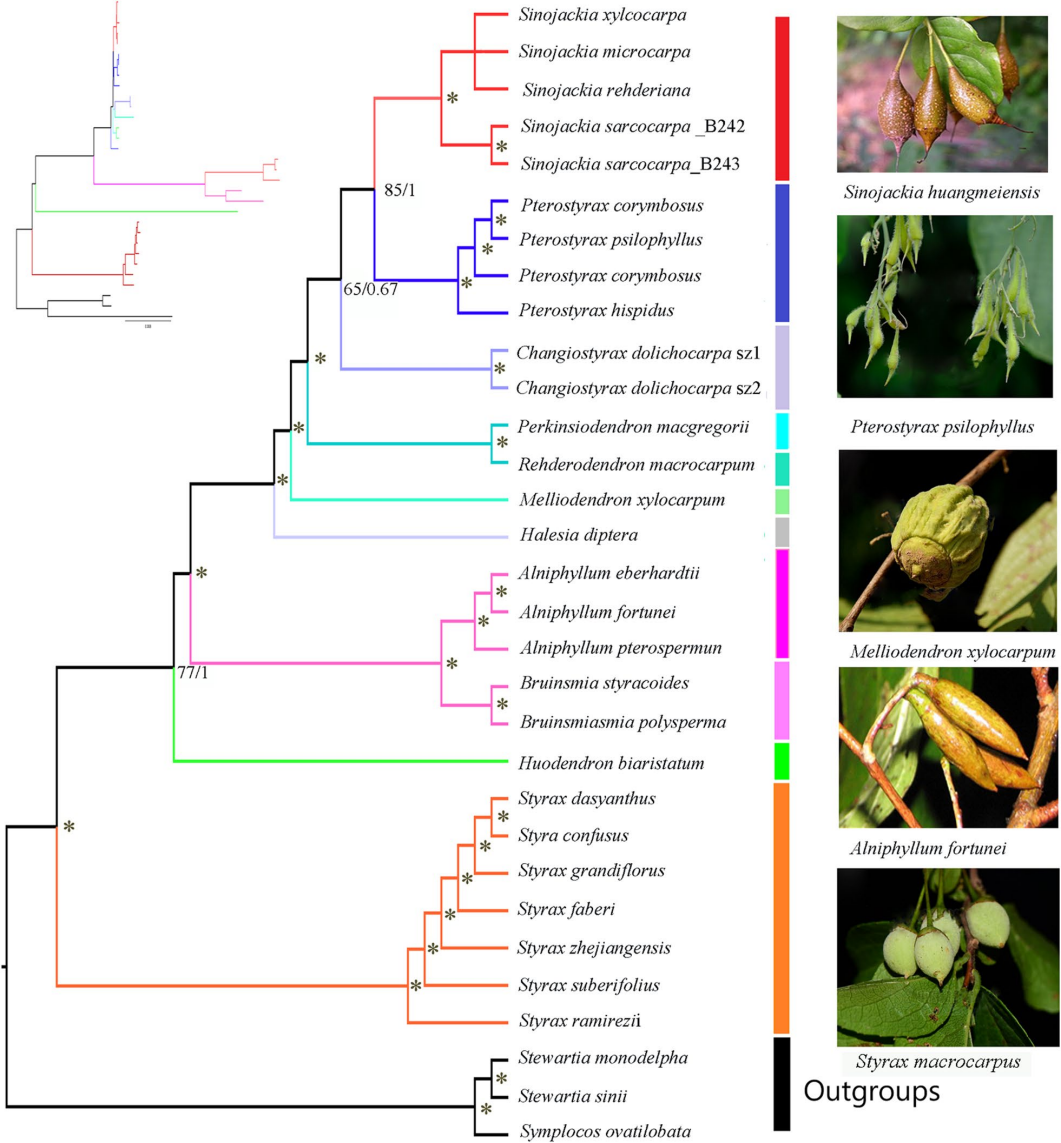
Optimal phylogenetic tree resulting from analyses of 79 protein-coding genes using Maximum Likelihood (ML). Bayesian inference (BI) topology is the same as ML. Support values next to the nodes are maximum likelihood bootstrap support/Bayesian posterior probability; asterisks indicate 100%/1.0 support values. The genera of Styracaceae are indicated by different branch colors. The inset shows the same tree as a phylogram

The optimal partitioning scheme identified under the Akaike information criterion with correction (AICc) using relaxed clustering analysis in PartitionFinder (lnL = − 18247.90; AICc = 379952.05) contained 64 partitions (Additional file 7: Table S1). BI analyses and ML analyses using the unpartitioned and partitioned schemes produced identical topologies (Fig. 6). Genera within Styracaceae were all recovered as monophyletic with strong support (BS/PP = 100/1). All species of *Styrax* form a clade sister to the rest of the family (BS/PP = 100/1). The second branch is *Huodendron*, followed by two genera with the 20-Kb inversion, *Alniphyllum* and *Bruinsmia*. *Halesia diptera* did not cluster with *Perkinsiodendron* but was sister to the remaining genera (BS/PP = 100/1), while *Perkinsiodendron* and *Rehderodendron* form a clade (BS/PP = 100/1). The position of *Melliodendron* does change based on the data partition analyzed. In most analysis *Melliodendron* is sister to a clade of *Perkinsiodendron, Rehderodendron, Changiostyrax, Pterostyrax,* and *Sinojackia* (BS/PP = 100/1) except for in LSC, which *Melliodendron* is sister to *Changiostyrax* form a clade (BS/PP = 56/1). *Changiostyrax* is sister to a clade composed of *Pterostyrax* and *Sinojackia* (BS/PP = 65/0.67). *Pterostyrax* and *Sinojackia* are sister with strong support (BS/PP = 85/1). To test for conflicting signals across different data, we used six data sets for analyses (Additional files 1, 2, 3, 4, 5, 6: Fig. S1–S6). The ML and BI analyses produced similar topologies over all data sets except for the different positions of *Sinojackia sarcocarpa* (L.) Q. Luo, *Changiostyrax dolichocarpus* (C. J. Qi) Tao Chen and *Pterostyrax hispidus* in the IR regions (Additional file 1: Fig S1). In trees inferred from the IR regions, *Sinojackia* and *Pterostyrax* were not monophyletic. Characteristics of all data sets are shown in Table 2.

## Discussion

### Plastome structure comparisons and sequence divergence hotspots

This study included 31 plastomes, 28 representative taxa from 11 genera of Styracaceae, and three outgroups. Plastomes displayed a typical quadripartite structure and similar size, containing a pair of inverted repeat IR regions (IRa and IRb), one large single-copy (LSC) region, and one small single-copy (SSC) region. The plastome size of Styracaceae is within the normal range of angiosperms (120–190 kb), and the size, structure, gene sequence and content of the whole family are highly conserved (155,185 bp–158,879 bp), with a typical tetragonal structure [54]. The plastome of *Alniphyllum fortunei*, which was first reported in this study, contained a 20-kb inversion which includes 14 coding genes from *trnQ-UUG* to *rpoB*. The presence of this inversion has previously been verified using PCR and Sanger sequencing by Yan et al. [55]. The inversion has also been observed in plastomes of *A. eberhardtii* Guill, *A. pterospermum* Matsum*, Bruinsmia polysperma* (C. B. Clarke) Steenis and *B. styracoides* Boerl. & Koord*,* suggesting that the inversion is common to *Bruinsmia* and *Alniphyllum*. The large 20-kb inversion has the same gene composition and relative position as the normal plastome structure and is not due solely to the genome assembly method [55]. Plastid structure is usually conserved in most angiosperms, but large inversions have been detected in many taxa. For example, a 4-kb inverted fragment in the LSC between *rpoB-trnT* was found in *Myriophyllum spicatum* [56], and a large gene inversion has also found in *Lotus japonicas*, *Arabidopsis thaliana* [57] and members of Oleaceae [58]. Because of their scarcity, plastid inversions are of great value to the study of genome evolution [59, 60]. Previous studies have suggested that gene inversions are closely related to repetitive sequences, and dispersed repetitive sequences promote inversions through intermolecular recombination [61–63].

In the sequence divergence analysis, the variation in loci of noncoding regions is higher than those of coding regions, which is similar to previous results of most angiosperms [64–66]. The results also show that the degree of evolution in the noncoding regions is greater than that of coding regions, and highly variable noncoding regions are of great value for the study of plant phylogenetics [67, 68]. In addition, the rate of variation in the IR region was lower than the two single copy regions. Previous studies have shown that the accumulation of point mutations in the inverted repeat region is slower than the single copy region [69–71].

### Positive selection analysis

In the selection pressure analysis, Styracaceae is dominated by synonymous substitutions. A previous study indicated that the rate of nonsynonymous substitutions is positively correlated with the degree of variation in the genome, while the rate of synonymous substitution exhibits a weak correlation with the degree of variation in the genome [72]. There are seven coding genes under positive selection, including five gene types: NADH dehydrogenase gene (*ndhH*), ribosomal protein coding gene (*rps4* & *rpl23*), RNA polymerase gene (*rpoC*1 & *rpoA*), a photosynthetic gene (*psaA*) and one additional protein gene (*accD*). The chloroplast NADH dehydrogenase (NDH) complex participates in the circular electron transport and chlorine respiration around the light system [73]. However, due to NDH complex existing in low abundance and being of a fragile nature, it is difficult to analyze its function [74]. The plants of Styracaceae are mainly distributed in the tropics and subtropics, which are subjected to growing conditions of high light and high temperature. Ribosomal proteins are a part of the ribosomal complex, which is a translation mechanism, and is essential for the correct production of proteins required for normal cell function. The selection of ribosomal proteins may increase the stability of ribosomal complexes under high light conditions, as well as high temperature, which is similar to the selection of *ndh* proteins under high light conditions [75]. However, whether these ribosomal proteins have increased stability over those of the original proteins under strong light or related conditions has not been determined, and further experimental verification is still needed. The *rpoC* gene is in the same operon as *rpoA*, which encodes the β subunit of RNA polymerase. Increasing the *rpoA* & *rpoC* mutations may lead to alterations in cell wall metabolism, possibly as a result of altered transcription [76]

### Phylogenetic analyses

We constructed data matrices from seven different partitions, and analyzed the phylogeny of the different matrices to maximize the resolution of phylogenetic relationships and to test for conflicting signals. Overall, the phylogenetic relationships constructed by the different data matrices show consistent topologies with moderate support. The phylogeny based on the complete plastome is consistent with the inferred phylogenies of the other six data sets with the exception of the IR region. According to Fritsch et al.’s [1] analysis of morphology and three DNA sequence data sets, *Styrax* is monophyletic, forming a clade with *Huodendron*. However, our analyses show that *Styrax* is monophyletic with high support (BS/PP = 100/1) but is sister to the remainder of the family, which is consistent with the conclusions of Yan et al. [27]. *Alniphyllum* and *Bruinsmia* formed a clade that has the longest branches in the phylogram, which may be due to higher rates of substitution in these two genera.

Fritsch et al. [1] and Yao et al. [26] consistently showed that *Melliodendron* formed a clade with *Changiostyrax*, whereas in all our data sets, except in the LSC data set, *Melliodendron* and *Changiostyrax* do not form a clade. *Changiostyrax* is weakly supported as sister to a clade composed of *Pterostyrax* and *Sinojackia* (BS/PP = 65/0.67). *Halesia* and *Pterostyrax* have not previously been fully resolved [1, 26, 27]. Here, we collected four accessions of *Pterostyrax* to analyze and *Pterostyrax* was recovered as monophyletic in all analyses except when *P. hispidus* was observed as being excluded from the other two species with a relatively low support value (BS/PP = 56/1) in the IR data set. Our study only included one species of *Halesia*, and its systematic relationship needs to be further verified by increasing the sample size or combining with nuclear gene analysis. *Perkinsiodendron* and *Rehderodendron* form a clade in our all data sets, with *Perkinsiodendron* being established as a new genus from *Halesia macgregorii* Chun based on molecular data and morphological characters [22]. Furthermore, our study strongly supports the monophyly of *Sinojackia* based on plastid data, as has been detected in previous studies [26], except in the IR data set where *Sinojackia sarcocarpa* is separated from the other species (BS/PP = 71/1). The different topological structure of the IR data set may be the result of a slower mutation and evolution rate compared to that of the single copy region [69–71, 77]. There are many possible reasons for differences between data sets in inferring phylogenetic trees, including taxonomic sampling and biological factors such as hybridization/introgression, incomplete lineage sorting, gene duplication and/or loss, and horizontal gene transfer [78–80]. However, most of these reasons do not explain differences observed between different partitions of complete plastome sequences. The conflicting signal from different partitions of the chloroplast may be caused by homoplasy rather than hybridization [1].

## Conclusions

Our results presented here utilize a phylogenomic data set to investigate phylogenetic relationships among the genera of Styracaceae. Based on 28 complete plastomes, our results show that the plastome structure of Styracaceae have small differences except for *Alniphyllum* and *Bruinsmia*, which have an approximately 20-kb inversion. Based on our almost complete species sampling for all genera except *Styrax*, all genera of Styracaceae are monophyletic, and the establishment of *Perkinsiodendron* and *Changiostyrax* are supported. Nevertheless, the lack of sequence data for species of *Parastyrax* necessitates that our results need to be further verified by increasing taxon sampling or population level sampling. With the increased sampling of taxa we can more effectively use the characteristics of faster evolving loci for phylogenetic inference [81, 82].

## Supplementary Information

**Additional file 1: Fig. S1.** Bayesian inference (BI) and Maximum likelihood (ML) phylogram of Styracaceae based on LSC regions, with ambiguous sites excluded from analysis. The support values on the branches are bootstrap value/Bayesian posterior probability; “*”means 100%/1.0 support values. The genera of Styracaceae are indicated by different colors, which correspond to branch colors.

**Additional file 2: Fig S2.** Bayesian inference (BI) and Maximum likelihood (ML) phylogram of Styracaceae based on SSC regions, with ambiguous sites excluded from analysis. The support values on the branches are bootstrap value/Bayesian posterior probability; “*”means 100%/1.0 support values. The genera of Styracaceae are indicated by different colors, which correspond to branch colors.

**Additional file 3: Fig. S3.** Bayesian inference (BI) and Maximum like- lihood (ML) phylogram of Styracaceae based on IR regions, with ambiguous sites excluded from analysis. The support values on the branches are bootstrap value/Bayesian posterior probability; “*”means 100%/1.0 support values. The genera of Styracaceae are indicated by different colors, which correspond to branch colors.

**Additional file 4: Fig. S4.** Bayesian inference (BI) and Maximum like-lihood (ML) phylogram of Styracaceae based on complete plastome sequences, with ambiguous sites excluded from analysis. The support values on the branches are bootstrap value/Bayesian posterior probability; “*”means 100%/1.0 support values. The genera of Styracaceae are indicated by different colors, which correspond to branch colors.

**Additional file 5: Fig. S5.** Bayesian inference (BI) and Maximum likelihood (ML) phylogram of Styracaceae based on plastome LSC+SSC regions, with ambiguous sites excluded from analysis. The support values on the branches are bootstrap value/Bayesian posterior probability; “*”means 100%/1.0 support values. The genera of Styracaceae are indicated by different colors, which correspond to branch colors.

**Additional file 6: Fig. S6.** Bayesian inference (BI) and Maximum likelihood (ML) phylogram of Styracaceae based on plastome noncoding regions, with ambiguous sites excluded from analysis. The support values on the branches are bootstrap value/Bayesian posterior probability; “*”means 100%/1.0 support values. The genera of Styracaceae are indicated by different colors, which correspond to branch colors.

**Additional file 7: Table S1.** The results of partitionfinder models in the study.

## Data Availability

All sequences used in this study are available from the National Center for Biotechnology Information (NCBI) (Accession Numbers: MT700470-MT700481; see Additional file 7: Table S1) .

## Abbreviations

BI: Bayesian Inference
CTAB: Cetyltrimethylammonium bromide
dN: Nonsynonymous
DnaSP: DNA Sequences Polymorphism
dS: Synonymous
IR: Inverted repeat
LSC: Large single copy
GTR: General time reversible
ML: Maximum Likelihood
PI: Phylogenetic informativeness
rRNA: Ribosomal RNA
SSC: Small single copy
tRNA: Transfer RNA
