# Supplementary figures and images for: Plastome structure and phylogenetic relationships of Styracaceae (Ericales)
Source: BMC Ecol Evol. 2021 May 28;21:103. doi: 10.1186/s12862-021-01827-4 (PMC8161964; doi:10.1186/s12862-021-01827-4)

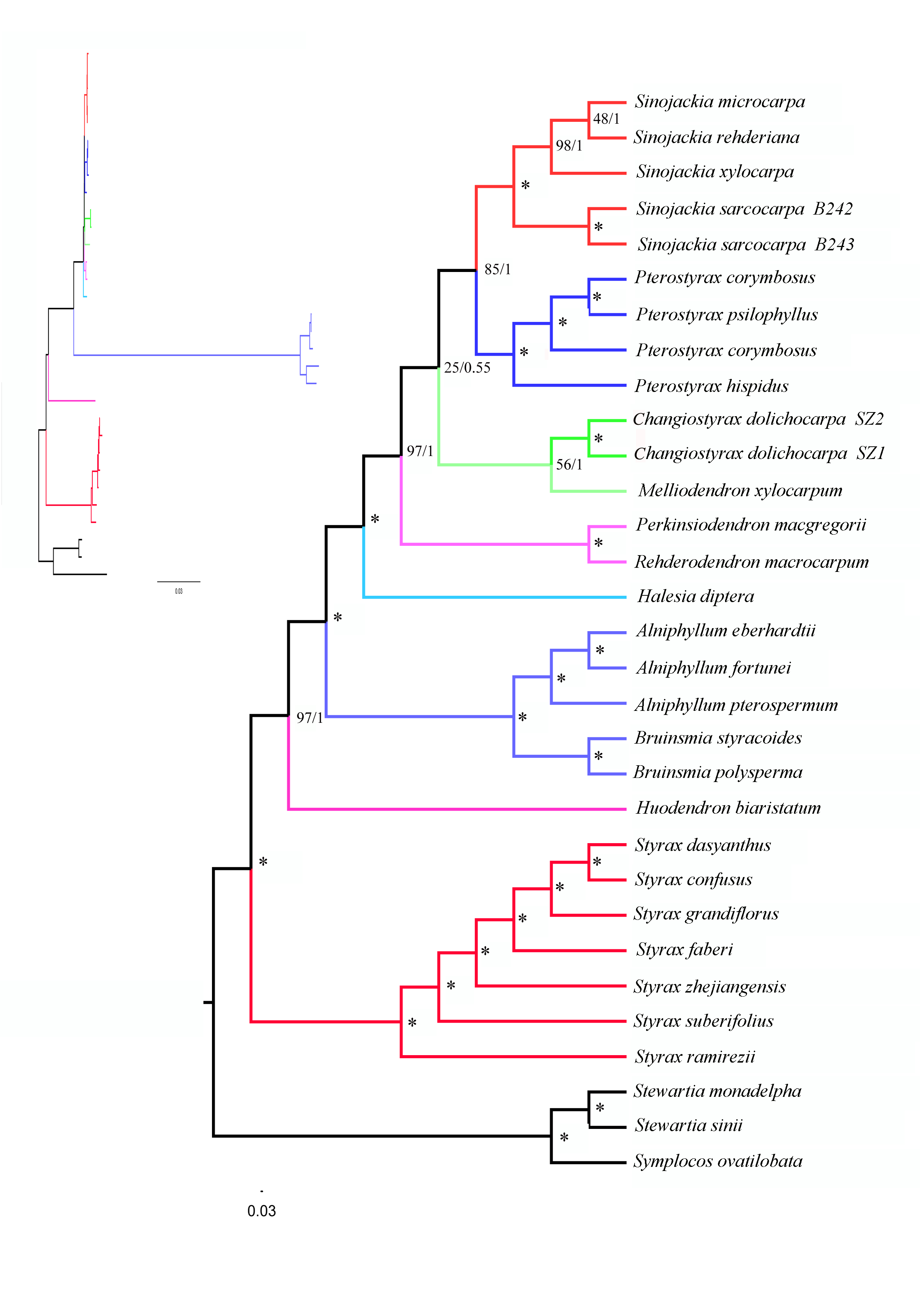

Supplement: Supplementary file 1 — Additional file 1: Fig. S1. Bayesian inference (BI) and Maximum likelihood (ML) phylogram of Styracaceae based on LSC regions, with ambiguous sites excluded from analysis. The support values on the branches are bootstrap value/Bayesian posterior probability; “*”means 100%/1.0 support values. The genera of Styracaceae are indicated by different colors, which correspond to branch colors. [file 12862_2021_1827_MOESM1_ESM.jpg]

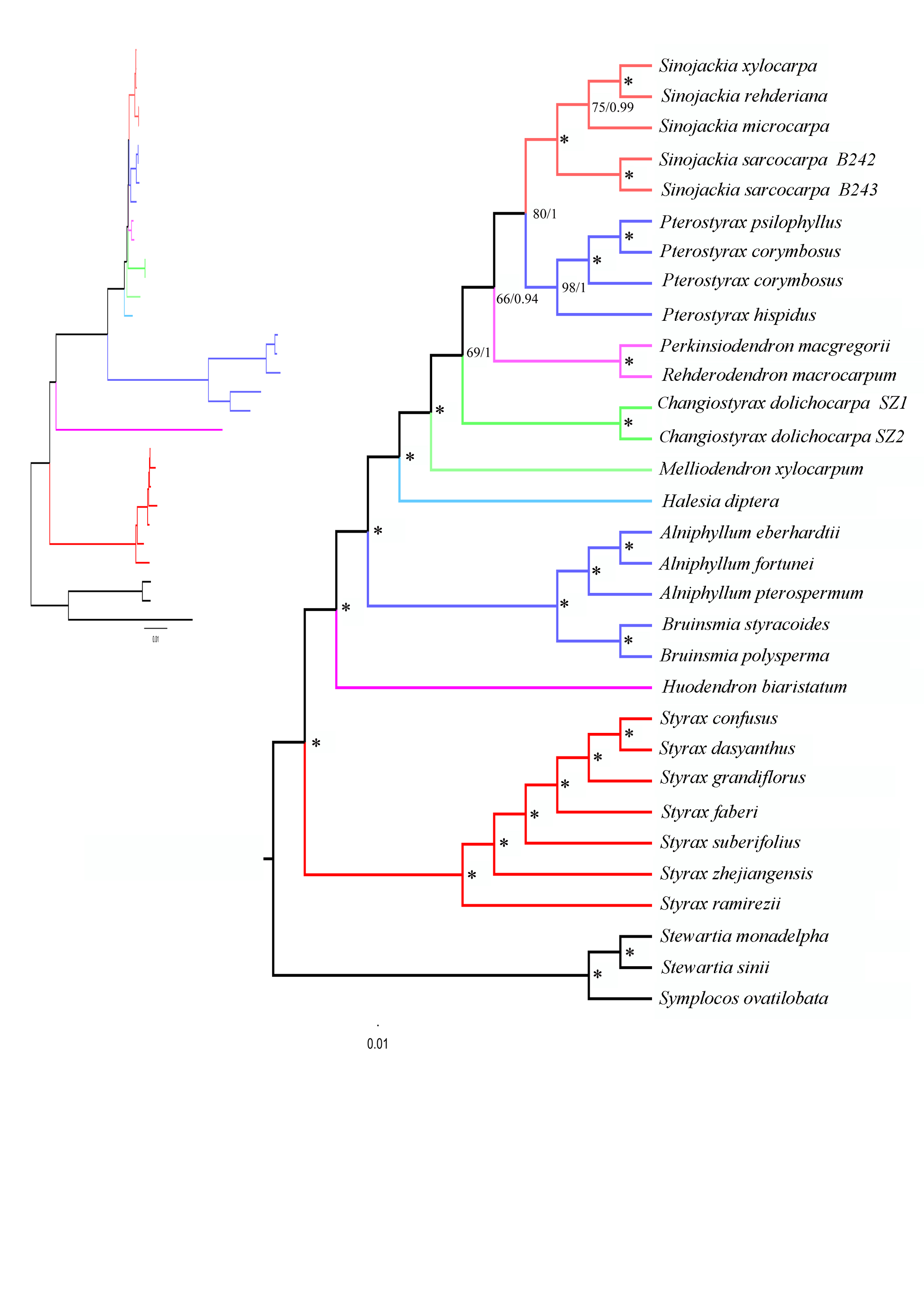

Supplement: Supplementary file 2 — Additional file 2: Fig S2. Bayesian inference (BI) and Maximum likelihood (ML) phylogram of Styracaceae based on SSC regions, with ambiguous sites excluded from analysis. The support values on the branches are bootstrap value/Bayesian posterior probability; “*”means 100%/1.0 support values. The genera of Styracaceae are indicated by different colors, which correspond to branch colors. [file 12862_2021_1827_MOESM2_ESM.jpg]

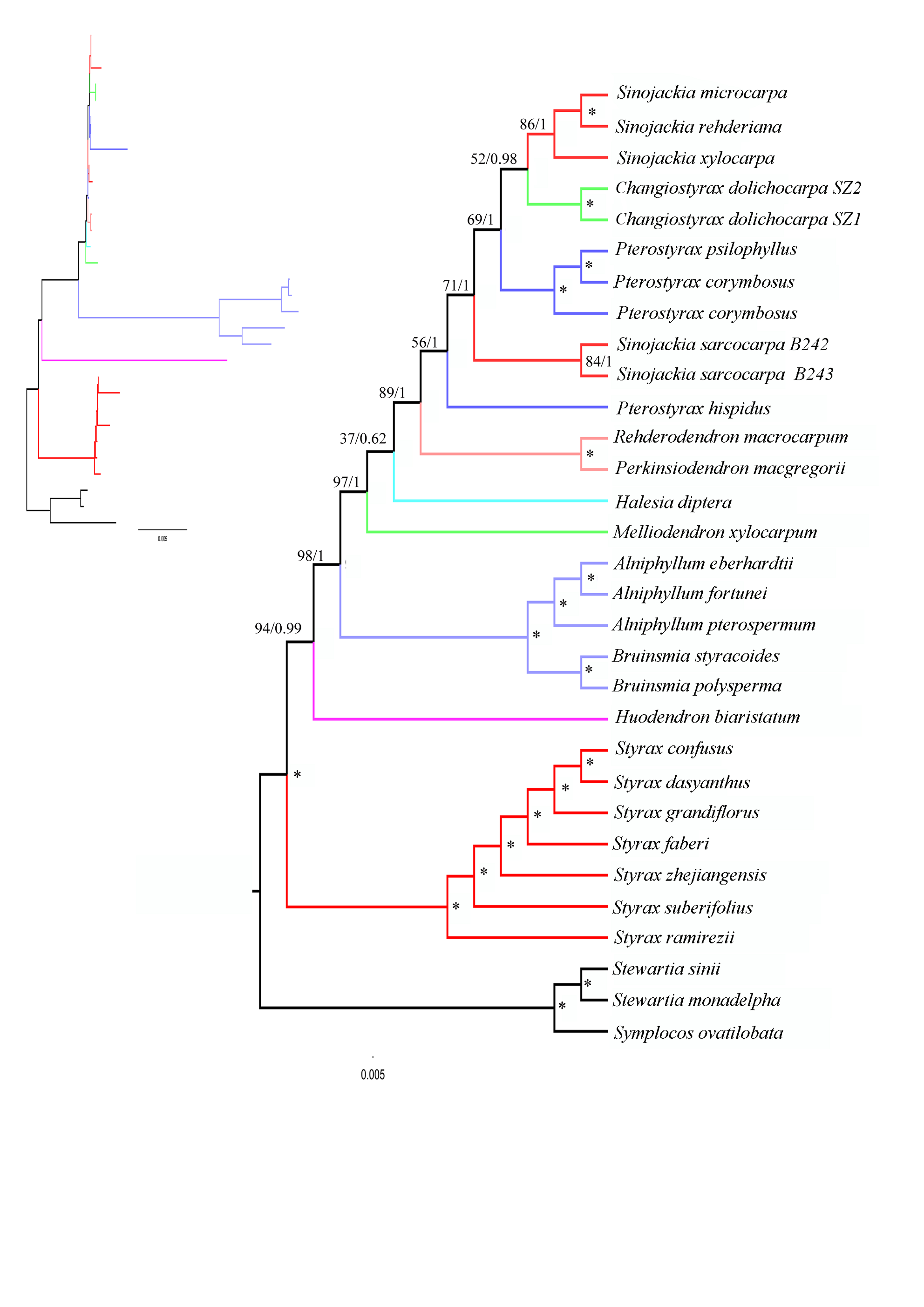

Supplement: Supplementary file 3 — Additional file 3: Fig. S3. Bayesian inference (BI) and Maximum like- lihood (ML) phylogram of Styracaceae based on IR regions, with ambiguous sites excluded from analysis. The support values on the branches are bootstrap value/Bayesian posterior probability; “*”means 100%/1.0 support values. The genera of Styracaceae are indicated by different colors, which correspond to branch colors. [file 12862_2021_1827_MOESM3_ESM.jpg]

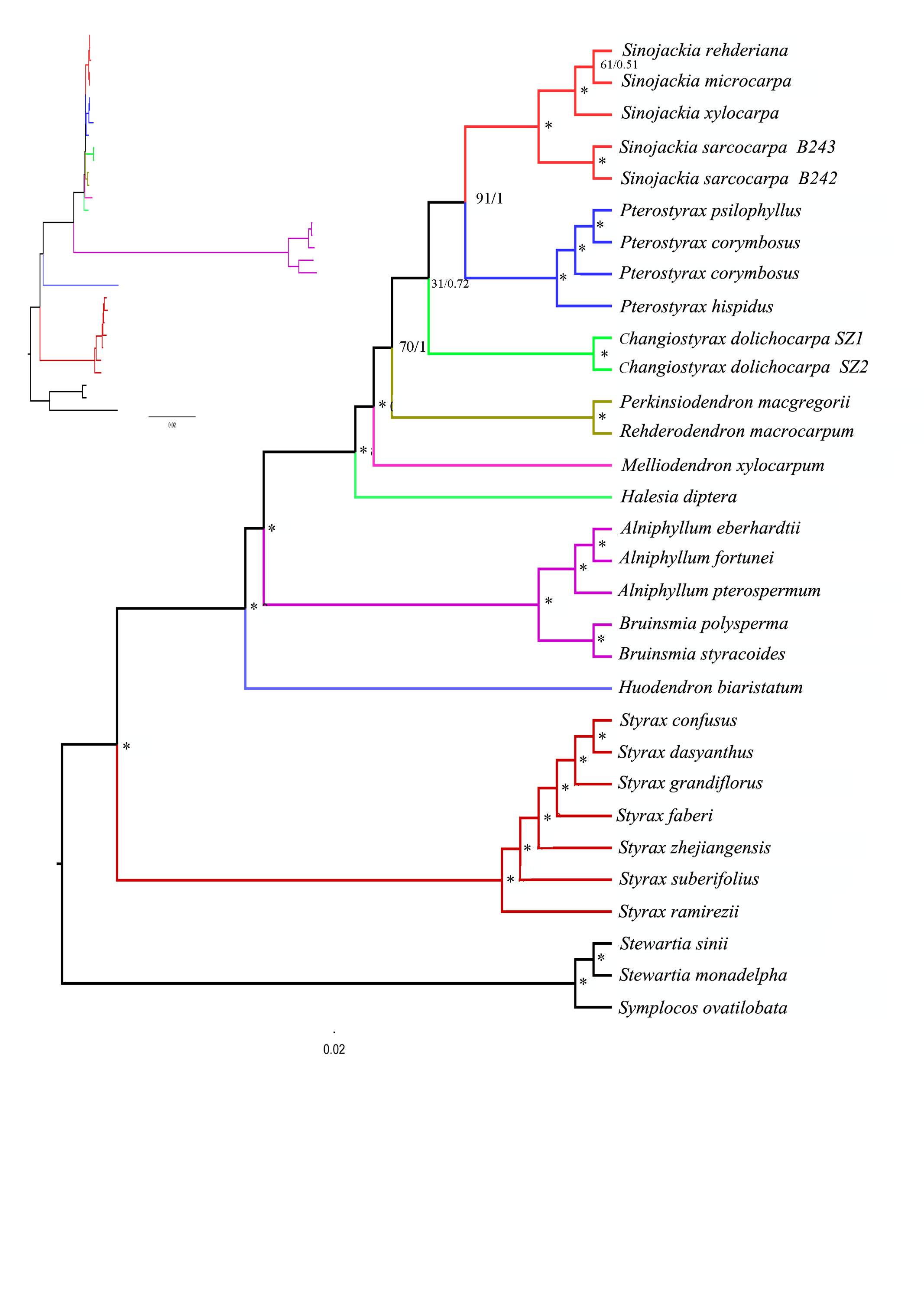

Supplement: Supplementary file 4 — Additional file 4: Fig. S4. Bayesian inference (BI) and Maximum like-lihood (ML) phylogram of Styracaceae based on complete plastome sequences, with ambiguous sites excluded from analysis. The support values on the branches are bootstrap value/Bayesian posterior probability; “*”means 100%/1.0 support values. The genera of Styracaceae are indicated by different colors, which correspond to branch colors. [file 12862_2021_1827_MOESM4_ESM.jpg]

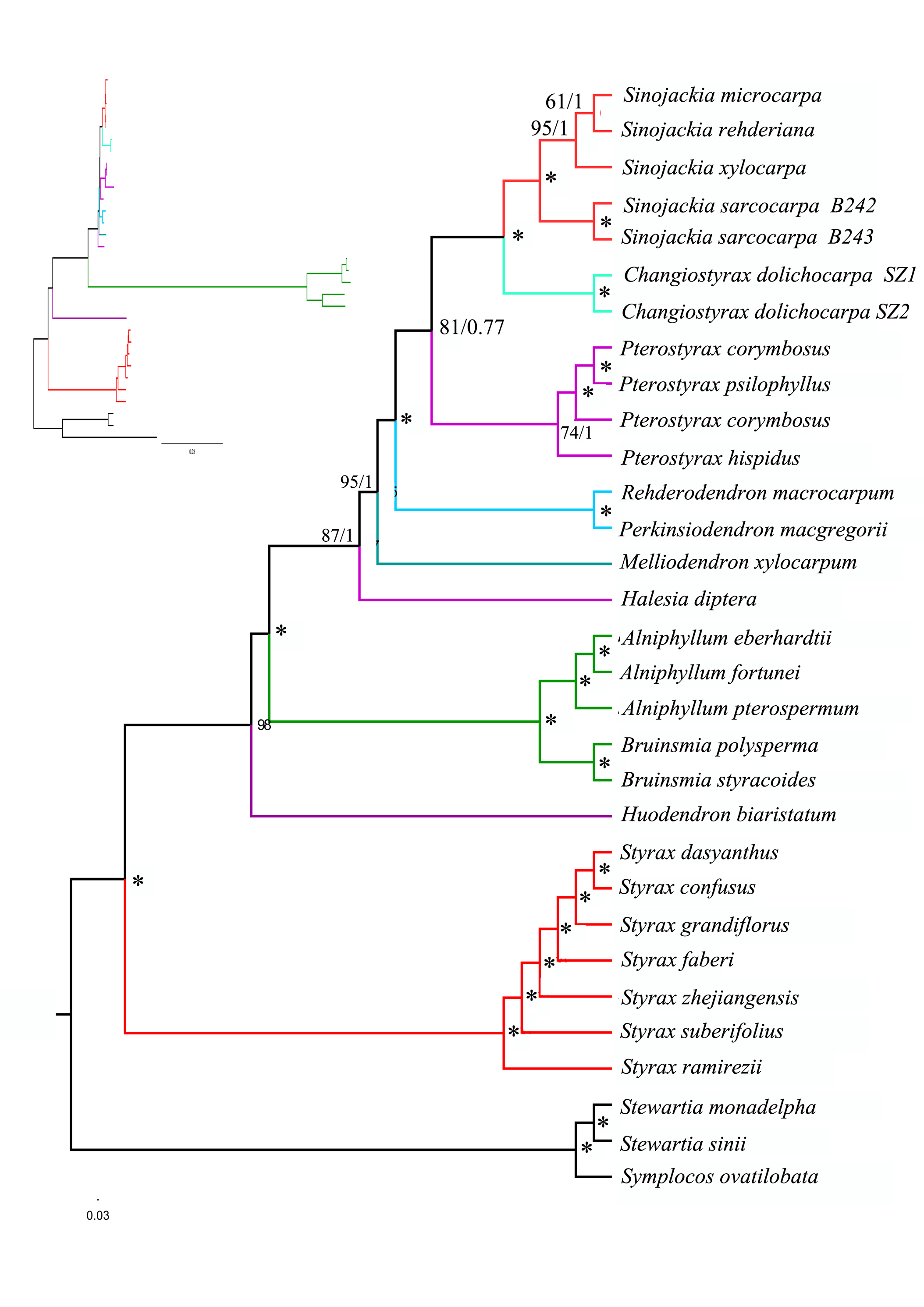

Supplement: Supplementary file 5 — Additional file 5: Fig. S5. Bayesian inference (BI) and Maximum likelihood (ML) phylogram of Styracaceae based on plastome LSC+SSC regions, with ambiguous sites excluded from analysis. The support values on the branches are bootstrap value/Bayesian posterior probability; “*”means 100%/1.0 support values. The genera of Styracaceae are indicated by different colors, which correspond to branch colors. [file 12862_2021_1827_MOESM5_ESM.jpg]

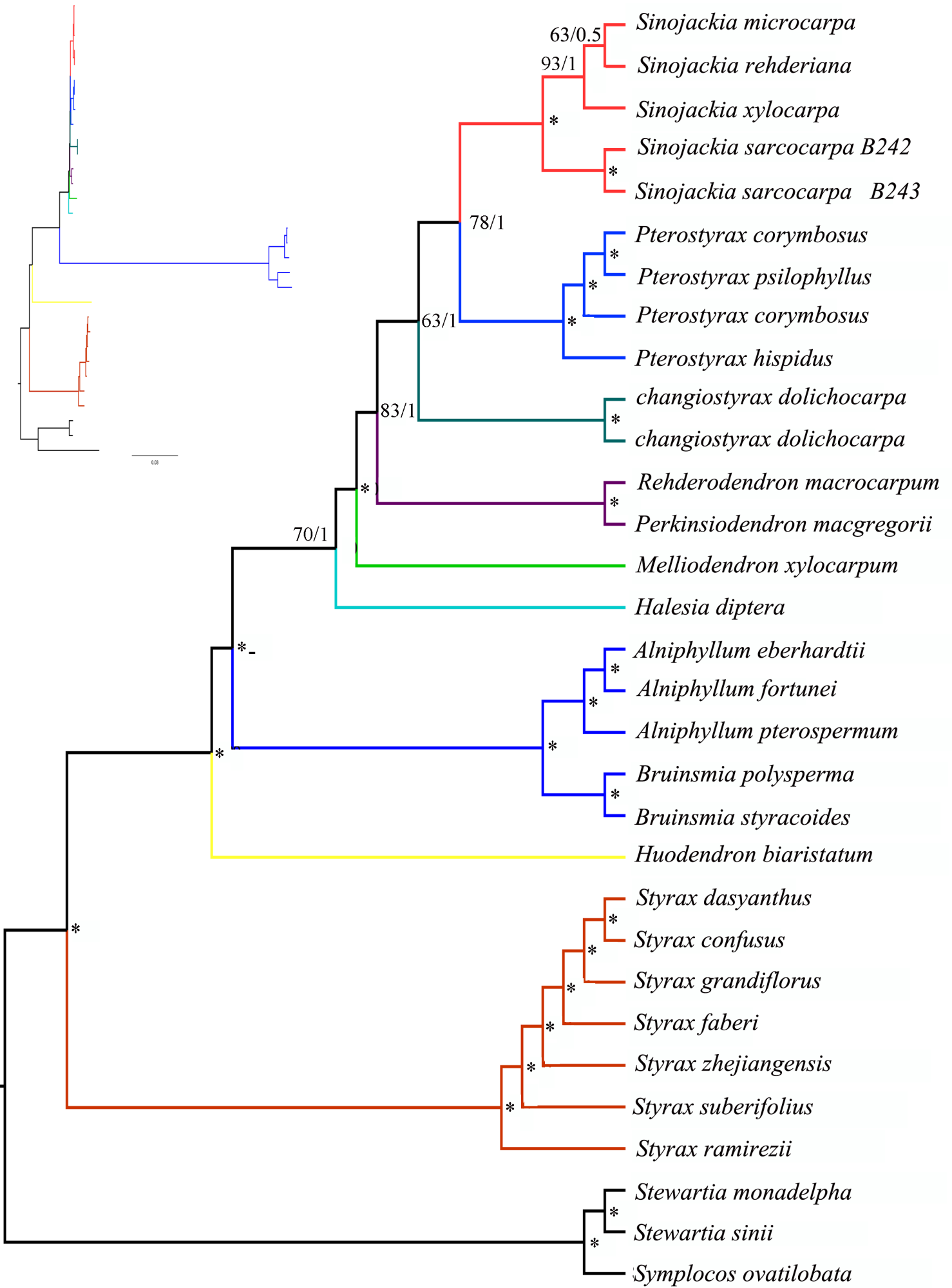

Supplement: Supplementary file 6 — Additional file 6: Fig. S6. Bayesian inference (BI) and Maximum likelihood (ML) phylogram of Styracaceae based on plastome noncoding regions, with ambiguous sites excluded from analysis. The support values on the branches are bootstrap value/Bayesian posterior probability; “*”means 100%/1.0 support values. The genera of Styracaceae are indicated by different colors, which correspond to branch colors. [file 12862_2021_1827_MOESM6_ESM.pdf]
