# Supplementary material for: Plastome structure and phylogenetic relationships of Styracaceae (Ericales)
Source: BMC Ecol Evol. 2021 May 28;21:103. doi: 10.1186/s12862-021-01827-4 (PMC8161964; doi:10.1186/s12862-021-01827-4)
Supplement: Supplementary file 7 — Additional file 7: Table S1. The results of partitionfinder models in the study. [file 12862_2021_1827_MOESM7_ESM.pdf]

Table S1. The results of partitionfinder models in the study.

Subset	Best Model	Partition names
1	GTR+G	accD_pos1
2	GTR+G	accD_pos2
3	GTR+G	accD_pos3
4	GTR+G	atpA_pos1, petA_pos1, rpl16_pos1
5	GTR+I+G	atpA_pos2, rpl32_pos2, ndhI_pos1, rpoB_pos2, ndhK_pos2, ndhI_pos2
6	GTR+G	rpl32_pos3, rps19_pos3, rps15_pos3, ndhA_pos3, atpA_pos3, rpoA_pos2
7	GTR+I+G	rbcL_pos1, atpB_pos1
8	GTR+G	atpB_pos2, ndhH_pos2,ndhB_pos3, rps19_pos2, atpE_pos2, petA_pos2
9	GTR+G	atpB_pos3, petD_pos3, rpoC1_pos1
10	GTR	rps7_pos1, rps11_pos1, rpl14_pos1, atpE_pos1
11	GTR+G	psbN_pos3, psbT_pos3, rpl14_pos3, atpE_pos3, ndhK_pos3, rps2_pos3, ycf3_pos3, rps4_pos1
12	GTR+G	rpoB_pos1, atpF_pos1, rpl16_pos2, ndhJ_pos1, rps14_pos2, rpoC1_pos2
13	GTR+G	atpF_pos2, ycf2_pos2
14	GTR+G	psaA_pos1, rps15_pos2, atpF_pos3, rps7_pos3, rps3_pos2, rps16_pos2
15	GTR	psbB_pos1, atpH_pos1, petD_pos1, psbC_pos1
16	GTR	petL_pos2, petN_pos2, atpH_pos2, psbH_pos2, psbJ_pos2, psaJ_pos2, psbZ_pos2
17	GTR+G	atpH_pos3, ndhE_pos3, petB_pos3, psaJ_pos3, petA_pos3, rbcL_pos3, ndhC_pos3, ndhJ_pos3, petL_pos3
18	GTR+I+G	atpI_pos1, rps8_pos2, ndhE_pos1, rpl36_pos2, rpl2_pos2
19	GTR+I+G	ndhD_pos2, ndhA_pos2, atpI_pos2
20	GTR+G	ndhD_pos3, ccsA_pos3, rpl22_pos3, ndhI_pos3, atpI_pos3, matK_pos3, cemA_pos3, rps8_pos3, infA_pos3, ndhG_pos3, ycf4_pos3, rps18_pos3, psbM_pos3, petG_pos3
21	GTR+G	ccsA_pos1, ndhD_pos1, ndhA_pos1
22	GTR+G	ccsA_pos2, ndhF_pos2, psbD_pos3
23	GTR	rpl32_pos1, rpl2_pos3, infA_pos2, petN_pos3, psaI_pos3, rpoC1_pos3, cemA_pos1, psaI_pos1, ycf15_pos2
24	GTR+G	cemA_pos2,psbL_pos2
25	GTR+G	clpP_pos1
26	GTR+G	ycf1_pos1, clpP_pos2, ycf1_pos2
27	GTR+G	clpP_pos3

28	GTR+G	rpl33_pos2, rps2_pos1, infA_pos1, rps19_pos1, rps8_pos1, rps3_pos1
29	GTR+G	matK_pos2, matK_pos1
30	GTR+I+G	ndhB_pos1, psaA_pos2, ndhJ_pos2, rpl23_pos2
31	GTR+I+G	ndhB_pos2, ndhE_pos2, ndhC_pos2, psbF_pos2, petB_pos2, psbT_pos2, petD_pos2
32	GTR	ndhC_pos1, ycf4_pos2
33	GTR+G	rps16_pos1, ndhF_pos1, rpl20_pos2, rpl20_pos3
34	GTR+G	ndhF_pos3
35	GTR+G	ndhG_pos1, ycf2_pos1, rps18_pos2
36	GTR+G	psbM_pos2, psaI_pos2, psbA_pos3, ndhG_pos2, psbK_pos2
37	GTR+G	rps3_pos3, ndhH_pos1, rps11_pos3, rps16_pos3, rpl36_pos3, rpl16_pos3
38	GTR	ndhH_pos3
39	GTR	ycf15_pos3, ndhK_pos1, ycf4_pos1
40	GTR+I+G	psbA_pos1, petN_pos1, ycf3_pos1, psaC_pos1, petB_pos1, rps12_pos1
41	GTR	petG_pos1, psbL_pos3, psaJ_pos1, psbT_pos1
42	GTR+I+G	psbI_pos1, petG_pos2, psbI_pos2
43	GTR+I+G	rpl23_pos1, ycf3_pos2, petL_pos1, rps12_pos3
44	GTR+I+G	psaA_pos3
45	GTR	psbN_pos1, psbD_pos1, psaB_pos1, psbE_pos1, psbF_pos1
46	GTR+I+G	psbE_pos2, psbB_pos2, psaB_pos2, psbC_pos2, psbA_pos2, psbD_pos2, psbL_pos1, psbN_pos2
47	GTR+G	psbE_pos3, psbC_pos3, psaB_pos3, psbB_pos3, rpl33_pos3
48	GTR	rps12_pos2, rps7_pos2, psaC_pos2, psbM_pos1,
49	GTR+G	psbH_pos3, psaC_pos3
50	GTR	rpl23_pos3, psbZ_pos1, psbJ_pos1, psbH_pos1, rpl14_pos2, rpl33_pos1, psbF_pos3
51	GTR	psbI_pos3, psbK_pos3, psbJ_pos3, psbZ_pos3
52	GTR+G	psbK_pos1, rps4_pos2, rpoC2_pos2, rps2_pos2
53	GTR+G	rbcL_pos2
54	GTR+G	rpl2_s_pos1, rpl2_pos1
55	GTR+G	rps18_pos1, rpl20_pos1, rps15_pos1, rpl22_pos2
56	GTR+G	rpl22_pos1, rps14_pos3
57	GTR+I+G	rpl36_pos1, rps14_pos1, ycf15_pos1
58	GTR+G	rpoA_pos1
59	GTR+G	rpoA_pos3
60	GTR+G	rpoC2_pos3, rpoB_pos3
61	GTR+G	rpoC2_pos1

62	GTR+G	rps11_pos2, rps4_pos3
63	GTR+G	ycf1_pos3
64	GTR+G	ycf2_pos3
